# MAFLD in Patients with Cushing's Disease Is Negatively Associated with Low Free Thyroxine Levels Rather than with Cortisol or TSH Levels

**DOI:** 10.1155/2023/6637396

**Published:** 2023-04-12

**Authors:** Kuangyang Chen, Lijiao Chen, Jiarong Dai, Hongying Ye

**Affiliations:** Department of Endocrinology and Metabolism, Huashan Hospital, Shanghai Medical College, Fudan University, Shanghai 200040, China

## Abstract

**Purpose:**

This study aims to analyze the clinical characteristic of metabolic associated fatty liver disease (MAFLD) in patients with active Cushing's disease (CD) and determine associations of thyroid hormones with MAFLD.

**Methods:**

Patients with active CD were included in this cross-sectional study. All subjects were assessed for hepatic steatosis by abdominal ultrasonography and thyroid functions. Demographic and clinical characteristic parameters were collected for correlation analysis and logistic analysis.

**Results:**

290 individuals with active CD were included in Huashan hospital from January 2014 to February 2022. We found that the prevalence of CD with MAFLD was 33.79%. The MAFLD group had a lower level of FT4 and a higher level of FT3/FT4 but no difference in levels of cortisol, 24 h UFC, TSH, TT4, TT3, and FT3. Correlation analysis showed positive associations of TSH, TT4, TT3, FT3, and FT3/FT4 with BMI. In age-, BMI-, sex-, cortisol-, and 24 h UFC-adjusted analysis, FT4 was independently associated with MAFLD in patients with CD. This association remained similar even after adjusting for the presence of metabolic syndrome components.

**Conclusion:**

Lower FT4 levels were associated with higher risk of MAFLD in patients with CD. FT4 may be used as a helpful indicator to predict MAFLD and provide novel ideas for the treatment of MAFLD in patients with CD in the future.

## 1. Introduction

Cushing's disease (CD), the most common cause of endogenous Cushing's syndrome (CS), is characterized by a lengthy and inappropriate exposure to excessive free glucocorticoids due to adrenocorticotropic hormone (ACTH) producing pituitary tumors [1]. Long-term excessive glucocorticoids influence the metabolism of glucose, protein, and lipids and thus lead to a complex metabolic syndrome, which consists of hypertension, abnormal glucose metabolism, dyslipidaemia, and obesity [2]. However, liver steatosis, regarded as the hepatic manifestation of metabolic syndrome and is associated with an increased risk of cardiovascular disease, has not been fully investigated in CD [3].

In addition to metabolic complications, excessive cortisol also leads to alterations in functions of the remaining endocrine hormone axes, including hypothalamic-pituitary-thyroid (HPT) axes. Usually, excessive cortisol inhibits the release of thyrotropin-releasing hormone (TRH) in the hypothalamus and thyroid stimulating hormone (TSH) in the pituitary, leading to a decrease in thyroxine (T4) and triiodothyronine (T3) in CS patients [4]. Thyroid functions would gradually normalize in most remitted or cured CD patients after transsphenoidal pituitary tumor resection [5]. In adrenal CS patients, serum TSH and free T4 levels were also decreased, and dysfunction of the hypothalamic-pituitary-thyroid axis was reversible after adrenalectomy [4].

It has been known that thyroid hormones are critical regulators for lipid metabolism by stimulating lipolysis in adipose tissues, regulating cholesterol metabolism, and increasing mitochondrial *β*-oxidation, autophagy, and the expression of genes involved in de novo lipogenesis in the liver [6]. It reports that patients in hypothyroidism had associated dyslipidemia and subsequently associated with nonalcoholic fatty liver disease (NAFLD) [7]. Mildly hypothyroid decreased insulin secretion and therefore suppressed the lipolysis response of adipose tissue to insulin and increased shuttling of fatty acids (FAs) to the liver, where they are esterified and accumulated as triglycerides [7]. However, characteristics of thyroid hormones in MAFLD in patients with CD were also rarely investigated.

Therefore, this cross-sectional study aims to evaluate the prevalence, biochemical characteristics, and risk/protective factors in CD patients with metabolic associated fatty liver disease (MAFLD) [8], especially exam associations of thyroid hormones with MAFLD in active CD.

## 2. Materials and Methods

### 2.1. Study Design

This was a cross-sectional study performed at the department of Endocrinology and Metabolism in Huashan Hospital. According to ICD-10, all subjects diagnosed to Cushing's disease and admitted to hospital treatment were included from January 2014 to February 2022. To avoid subjective bias, patients received detailed clinical examination after enrollment by the same group of endocrinology specialists. Our study was approved by the Ethics Committee of Huashan Hospital.

### 2.2. Participants and Definitions

We included patients diagnosed with Cushing's disease at the department of Endocrinology and Metabolism in 2014–2022. Cushing's disease was defined as follows [9]: (1) exclude excessive exogenous glucocorticoid exposure and physiologic causes; (2) conditions and clinical features of Cushing's syndrome; (3) an elevated levels of 24 h UFC (at least two measurements); (4) failure of plasma cortisol decreased after an overnight 1 mg dexamethasone suppression test; and (5) pituitary lesion >6 mm showed by a magnetic resonance imaging (MRI) scan or positive results in an inferior petrosal sinus sampling in patients with negative MRI images or a lesion <6 mm.

The definition of MAFLD was according to a consensus proposed by a panel of experts from 22 countries, namely, hepatic fat content >5% (diagnosed by abdominal ultrasonography here) and accompany with any one of the following conditions: (1) overweight or obesity, (2) presence of diabetes mellitus, and (3) or at least two metabolic risk abnormalities (waist circumference ≥90/80 cm in men or women; blood pressure ≥130/85 mmHg or drug treatment; TG ≥ 1.70 mmol/L or drug treatment; HDL-c < 1.0 mmol/L for men and <1.3 mmol/L for women or drug treatment; prediabetes; HOMA-IR ≥ 2.5; and high-sensitivity C-reactive protein >2 mg/L) [8]. Patients with active CD were categorized into two groups for further analysis according to whether they suffered from MAFLD.

Additional exclusion criteria were as follows: (1) after transsphenoidal surgery, radiation therapy or pharmacological therapy for pituitary tumor, (2) use of anti-thyroid drugs or thyroid hormone or a history of thyroid surgery, and (3) missing thyroid function test results.

### 2.3. Data Collection and Calculations

All data were obtained by reviewing the medical history in the electronic medical record system. Age, sex, weight, and height were asked or measured after registration. Serum aspartate aminotransferase (AST), alanine aminotransferase (ALT), gamma-glutamyl transferase (GGT), albumin, platelets, total triglycerides (TG), total cholesterol (TC), low density lipoprotein (LDL), high density lipoprotein (HDL), glycated hemoglobin (HbA1c), blood glucose, insulin, C-peptide, and morning cortisol were detected under fasting condition between 8.00-10.00 am. Collecting 24-hour urine for detecting 24 h urine-free cortisol (24 h UFC) was also needed. All subjects were assessed for thyroid function, namely, thyroid stimulating hormone (TSH), total thyroxine (TT4), total triiodothyronine (TT3), free thyroxine (FT4), free triiodothyronine (FT3), and abdominal ultrasonography. After a systematic examination, comorbidities of each patient were judged and recorded by specialists. The data about epidemiologic, clinical, and biochemical data were gathered by two researchers after carefully reviewing the patient files. All the hormones were tested by the same assay.

The body mass index (BMI) was calculated as weight (kg) divided by height squared (m^2^). Normal weight: BMI < 23.9 kg/m^2^, overweight: 24 ≤ BMI <27.9 kg/m^2^, and obesity: BMI ≥ 28 kg/m^2^.

### 2.4. Statistical Analysis

All statistical analyses were performed by two researchers and were carried out by using Statistical Package for Social Sciences (version 26.0, SPSS, Inc., IBM, USA). A two-tail*P* value <0.05 was regarded as statistically significant. Continuous variables are given as the mean ± standard deviation when normally distributed and as the median with an interquartile range when skewedly distributed. In contrast, categorical variables are presented as absolute number (percentage). The parametric Student's *t*-test was used to compare two groups for continuous variables, and the chi-squared test was used for categorical variables. 24 h UFC, TSH, AST, ALT, GGT, TG and HDL-c were log transformed before analysis. The associations of thyroid functions with BMI were examined by Pearson's correlation and then graphed as line regression curves. Odds ratios (ORs) are given with 95% confidence intervals by using univariable logistic analysis and multivariable logistic analysis adjusted for confounders.

## 3. Results

**Figure 1:**
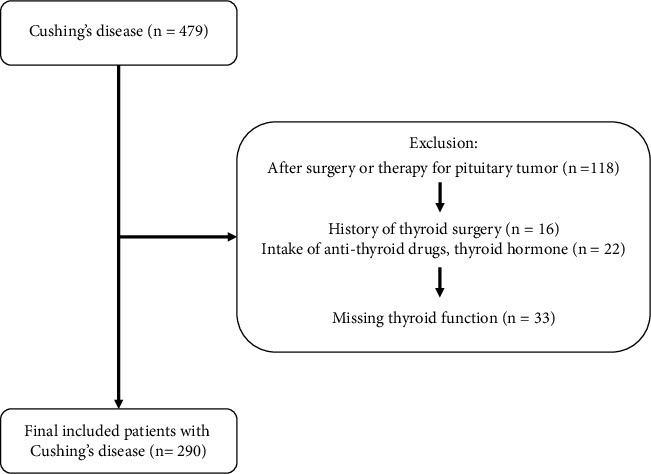
The flowchart of selection.

We originally selected 479 patients with Cushing's disease (CD), 34.66% of which had ultrasonography diagnosed hepatic steatosis, from the Department of Endocrinology and Metabolism in Huashan Hospital from January 2014 to February 2022. After applying the exclusion criteria, we finally included 290 patients with active CD in this study (as shown in Figure 1).

Among 290 enrolled patients with CD (mean age: 38.52 ± 12.84), more than thirty percent of patients had MAFLD (89/290, 33.79%). Females were more prone to suffer from CD when compared to males (78.28% versus 21.72%). 62.76% of patients with CD were overweight or obese (BMI > 24 kg/m^2^), 58.62% of patients had diabetes or impaired glucose tolerance, 73.10% of patients had hypertension, 30.00% of patients had hyperlipidemia, and 15.86% of patients had central hypothyroidism (FT4 below the lower reference bound without elevated TSH). Compared with patients without MAFLD, CD patients with MAFLD had significantly higher BMI (*P* < 0.001), percentage of hypertension (*P*=0.004), percentage of hyperlipidemia (*P*=0.002), AST (*P*=0.010), ALT (*P* < 0.001), GGT (*P* < 0.001), TG (*P* < 0.001), C-peptide (*P*=0.013) levels, and a lower HDL level (*P* < 0.001). No differences were found between two groups in the percentage of diabetes mellitus or impaired glucose tolerance, albumin, platelets, TC, LDL-c, fasting blood glucose (FBG), fasting insulin (FIN), 24 h free urine cortisol (24 h UFC), and morning cortisol levels as shown in Table 1 (All *P* > 0.05). Interestingly, serum FT4 levels were decreased and the FT3/FT4 ratio was increased in patients with MAFLD (both *P* < 0.05), whereas the levels of TSH, TT3, and FT3 had not difference (all *P* > 0.05). Among all subjects with CD, the percentages of subjects with TSH, TT4, TT3, FT4, and FT3 levels below the lower limit of reference range were 24.48%, 18.28%, 49.66%, 16.2%, and 32.07%, respectively. Further analyses showed that the percentage of patients with FT4 below the reference range was lower in MAFLD than that in non-MAFLD (*P*=0.040), but there were no differences in the percentage of patients with TSH, TT4, TT3, and FT3 below the normal range between two groups (all *P* > 0.05).

**Figure 2:**
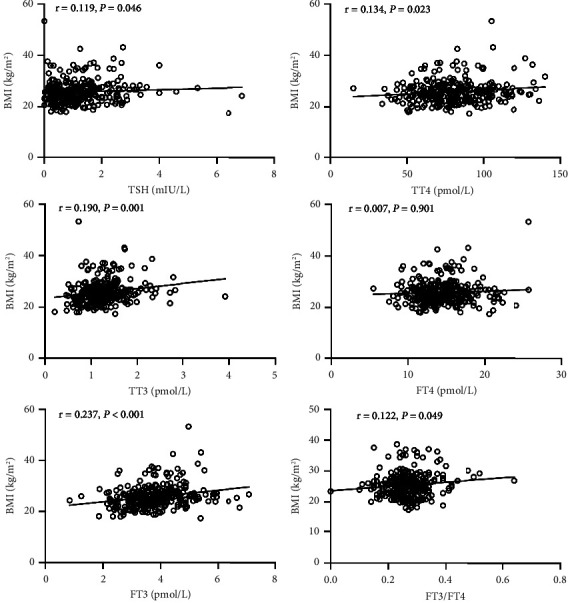
Correlation analysis of thyroid functions with the BMI in patients with Cushing's disease.

Correlations of the BMI and thyroid hormones were performed (as shown in Figure 2). TSH, TT4, TT3, FT3, and FT3/FT4 were positively correlated with the BMI (*r* = 0.119, *P*=0.046; *r* = 0.134, *P* < 0.023; *r* = 0.190, *P*=0.001; *r* = 0.237, *P* < 0.001; and *r* = 0.122, *P*=0.049, respectively). However, there was no significant association of FT4 with the BMI (*P* > 0.05).

We analyzed associations between hormones and MAFLD in patients with CD by logistic regression analyses (Table 2). In the crude model of univariate logistic regression, MAFLD in patients with CD was associated with the BMI (OR 1.228, 95% CI: 1.189–1.395, *P* < 0.001), sex (OR 1.794, 95% CI: 1.010–3.177, *P*=0.045), hypertension (OR 2.44495% CI: 1.321–4.522, *P*=0.004), and hyperlipidemia (OR 2.283, 95% CI: 1.357–3.842, *P*=0.002) but not with central hypothyroidism, age, cortisol, 24 h UFC, TSH, TT4, TT3, FT4, and FT3 (all *P* > 0.05). After adjusting age, BMI, sex, cortisol, 24 h UFC, TSH, TT4, TT3, FT4, and FT3, it showed that the BMI (OR 1.315, 95% CI: 1.197–1.444, *P* < 0.001), sex (OR 2.705, 95% CI: 1.229–5.955, *P*=0.013), and FT4 (OR 0.782, 95% CI: 0.647–0.946, *P*=0.011) were independently associated with MAFLD in patients with CD (Multivariate Model 1). In subsequent multivariate logistic regressions analysis including the presence of the individual MetS components (Multivariate Model 2), similar independent association of MAFLD with FT4 was also found (OR 0.770, 95% CI: 0.633–0.937, *P*=0.009).

Multivariate logistic regressions were performed to assess the associations between quartiles of thyroid hormones and MAFLD in patients with CD (Table 3). All patients were divided into 4 groups according to TSH, TT4, TT3, FT4, and FT3 quartiles, respectively. The first quartile was set to be the reference group. The ORs for the third and fourth quartile groups of FT4 were 0.257 and 0.220 (95% CI: 0.089–0.742, *P*=0.012; 95% CI: 0.059−0.824, *P*=0.014) as shown in Table 3 (the *P* value for trend = 0.018). Nevertheless, no different association was observed between MAFLD and TSH, TT4, TT3, or FT3 (all *P* > 0.05).

## 4. Discussion

This study firstly described the characteristic of MAFLD in CD and explored the associations between thyroid parameters and MAFLD in patients with CD. Among all patients with CD, the prevalence of MAFLD was 33.79%. We found that subjects with MAFLD in patients with CD had lower FT4 levels than subjects without. However, the TSH, FT3, and cortisol levels were similar between two groups. Furthermore, there was an independent association between lower FT4 and higher risk of MAFLD in patients with active CD.

Hepatic steatosis, affected nearly 25.24% people around the world and regarded as the hepatic manifestation of metabolic syndrome [10], was not taken into consideration frequently when referred to the comorbidities of CS. A Chinese retrospective study enrolled 1652 patients with CS and found that 27.4% patients had hepatic steatosis [11]. The prevalence of ultrasonography-diagnosed hepatic steatosis in all patients with CD (34.79%, 166/479) in this study was similar to the prevalence of hepatic steatosis in the normal population [10]. Although it is generally believed that glucocorticoids drive the development of NAFLD by acting on the liver and adipose tissue [12], no differences in morning cortisol and 24 h UFC between MAFLD and non-MAFLD groups in patients with CD were found in this study. A large epidemiological study also showed that the plasma cortisol had no association with hepatic steatosis in the urban population [13]. However, no correlation between MAFLD and serum cortisol, whether in patients with CD and the normal population does not rule out that glucocorticoids play an important role in the development of hepatic steatosis. In vivo and in vitro studies showed that glucocorticoids drive hepatic steatosis by promoting De Novo Lipogenesis (DNL), free fatty acid utilization, and reducing VLDL secretion in the liver and increasing delivery of fatty acid from adipose tissues into the liver [12]. However, the exact course of active CD before diagnosed in this study was unclear, which would influence the development of hepatic steatosis and the level of serum cortisol.

Hypothalamic-pituitary-thyroid axis is inhibited after long-term exposure to excess glucocorticoid to some extent [14]. We found that only a total of 94 (32.41%) had normal thyroid hormones. 15.86% patients with CD had secondary hypothyroidism, the percentage of which was lower than that in a Cushing's syndrome cohort (53%). The etiology and course of disease may contribute to the different prevalence. There is a reciprocal causation relationship between obesity and thyroid disorders [15, 16]. The BMI is associated with dysfunction of thyroid function especially primary hypothyroidism [17]. Although the associations between thyroid hormones and body weight status vary slightly in different studies, most available data showed that lower levels of FT4 or higher levels of FT3 or TSH were associated with a higher BMI in euthyroid subjects [18–21]. In primary hypothyroidism patients, TSH had no significant relationship with the BMI [22, 23]. In patients with subclinical hypothyroidism, the BMI was positively correlated with TSH and negatively correlated with FT4 after adjusting for age [24]. However, central hypothyroidism has been much less frequently studied because of its rarity. It reported that FT4 was also negatively associated with the BMI and waist circumference in growth hormone-deficient induced hypothyroidism patients [25]. In patients with CD, we found that lower levels of FT3 and TSH, but not FT4, were associated with a higher BMI. Usually, the divergent associations between the BMI and FT3 or FT4 would reflect an increased deiodinase activity [20]. However, patients with CD had an impaired deiodinase activity, which decreased the rate of conversion of T4 to T3 [26]. Moreover, muscle atrophy in CD patients also masks the relationship between the BMI and FT4 [27].

Numerous epidemiologists investigated the relationship between thyroid hormones and the incidence of the fatty liver. In a large population-based cohort study, a higher TSH level or lower FT4 was associated with a higher NAFLD risk [28]. A Lifeline cohort study considered 20289 euthyroid subjects and reported that NAFLD (FLI ≥ 60) was independently associated with a higher FT3 and a lower FT4 but not by TSH [29]. A meta-analysis reported that lower FT4 and higher TSH, but not FT3, had associations with the risk of NAFLD in primary hypothyroidism or subclinical hypothyroidism patients [30]. However, the association between MAFLD in patients with CD and thyroid hormones has not been investigated yet. Results in our study were partly similar to previous studies, namely an independent association of MAFLD and FT4 after adjusting confounders in patients with active CD, rather than TSH or FT3. After patients being divided into 4 groups according to TSH, TT4, TT3, FT4, and FT3 quartiles, respectively, the ORs for the third and fourth quartile groups of FT4 were negatively associated with MAFLD compared to the first quartile. A dose-response relationship was observed on FT4 quartile and MAFLD among patients with active CD. These findings were strengthened when similar associations were observed through various analytic strategies. Moreover, we found that cortisol-associated hypothyroidism had no association with MAFLD in patients with CD, which was distinct from the common view that primary hypothyroidism was highly associated with the fatty liver. Excessive glucocorticoid induced central hypothyroidism by reducing thyrotropin-releasing hormone expression and release in the hypothalamus, decreasing TSH secretion in pituitary, as well as inhibiting FT4 and FT3 levels. In Cushing's disease, the association between TSH and MAFLD might be blunted since there was not a relatively high TSH levels normally seen at a lower FT4 levels.

It is well known that thyroid hormones have prominent effects on hepatic fatty acid and cholesterol synthesis and metabolism [31]. As an active form of thyroid hormones, T3 has much greater thyroid hormone receptor affinity than T4 [32, 33]. Although higher FT3 levels were associated with higher risk of the fatty liver in several studies that were conducted in euthyroid subjects [29, 34, 35], it was FT4, but not fT3, that was associated with MAFLD in patients with CD in this cohort. High cortisol concentrations not only could inhibit TSH-stimulated T3 and T4 levels but also inhibit the conversion of T4 to T3, which made T3 levels in subjects more complex [36]. In addition, FT3 was susceptible to nutritional status or being sick. However, FT4 is more meaningful for the diagnosis of thyroid status, and the decreased FT4 concentration was used to mark the symptoms of hypothyroidism in patients with CD. Although the exact mechanisms are unclear, FT4 may be used as a helpful indicator to predict MAFLD and provide novel ideas for the treatment of MAFLD in patients with CD.

## 5. Limitation

The present study had several limitations. First, similar to other cross-sectional studies, causal correlation cannot be established with certainty in this study. The incidence or degree of hepatic steatosis was related to the duration of CD which was tough to evaluate in this study. Second, we did not analysis the relationship of thyroid hormones with MAFLD in active CD with euthyroid subjects or central hypothyroidism groups separately due to the limited statistical power. Third, the BMI in isolation is an insufficient biomarker of abdominal obesity, and waist circumference was recommended to be included in the evaluation of overweight and the obesity group [37]. Therefore, it would be better to investigate the association between thyroid hormones and obesity or MAFLD by using waist circumference or the whole-body MRI, which was a limitation of this study.

## 6. Conclusion

In conclusion, CD patients with MAFLD were associated with a lower level of FT4 rather than FT3 or cortisol. Theoretically, glucocorticoids do have direct and indirect actions on the development of hepatic steatosis, but it seemed that serum cortisol had no independent value in the diagnosis of MAFLD in patients with CD. Moreover, our results found that lower FT4 increased the risk of MAFLD in patients with CD. More attention should be paid to optimize the replacement therapy in the future and improve awareness of thyroid status, especially the level of FT4, to decrease the risk of MAFLD in patients with CD.

## Figures and Tables

**Table 1:** Demographics and biochemical characteristics in Cushing's disease patients with and without MAFLD.

Variables	All (*N* = 290)	Without MAFLD (*N* = 192)	MAFLD (*N* = 98)	*P* value
Demographic features				
Sex				
Male, *N* (%)	63 (21.72)	35 (18.23)	28 (28.57)	**0.043 ** ^a^
Female, *N* (%)	227 (78.28)	157 (81.77)	70 (71.43)	—
Age (years)	38.52 ± 12.84	38.61 ± 13.48	38.33 ± 11.10	0.911^b^
BMI (kg/m^2^)	25.83 ± 4.51	24.45 ± 3.26	28.51 ± 5.36	**<0.001 ** ^b^
<24, *N* (%)	108 (37.24)	93 (48.43)	15 (15.31)	**<0.001 ** ^a^
≥24, *N* (%)	182 (62.76)	99 (51.56)	83 (84.69)	—
Comorbidities				
Diabetes/IGT, *N* (%)	170 (58.62)	108 (56.25)	62 (63.27)	0.251^a^
Hypertension, *N* (%)	212 (73.10)	130 (67.70)	82 (83.67)	**0.004 ** ^a^
Hyperlipidemia, *N* (%)	87 (30.00)	46 (25.52)	41 (41.84)	**0.002 ** ^a^
Central hypothyroidism, *N* (%)	46 (15.86)	26 (13.54)	20 (20.4)	0.130^a^
Biochemistry assays				
AST (IU/L)^#^	17.00 (14.00–21.00)	17.00 (14.00–19.25)	18.50 (15.00–25.00)	**0.010 ** ^b^
ALT (IU/L)^#^	26.00 (18.00–40.00)	22.00 (16.00–32.25)	29.50 (22.00–58.25.00)	**<0.001 ** ^b^
GGT (IU/L)^#^	27.00 (18.75–44.00)	22.50 (16.00–31.25)	38.00 (25.00–62.75)	**<0.001 ** ^b^
Albumin (g/L)	42.69 ± 4.29	43.11 ± 3.97	43.69 ± 3.73	0.282^b^
PLT (10^^9^/L)	237.79 ± 67.75	229.39 ± 56.45	244.17 ± 76.69	0.200^b^
TC (mmol/L)	4.70 ± 1.74	4.79 ± 1.66	4.59 ± 1.91	0.505^b^
TG (mmol/L)^#^	1.32 (0.94–2.01)	1.24 (0.90–1.70)	1.68 (1.13–2.72)	**<0.001 ** ^b^
HDL-c (mmol/L)^#^	1.41 (1.17–1.74)	1.45 (1.24–1.87)	1.28 (1.10–1.55)	**<0.001 ** ^b^
LDL-c (mmol/L)	2.96 ± 1.17	2.86 ± 1.09	3.07 ± 1.21	0.250^b^
HbA1c (%)	6.24 ± 1.15	6.03 ± 1.11	6.38 ± 1.14	0.099^b^
FBG (mmol/L)	6.07 ± 2.85	6.06 ± 3.58	6.36 ± 2.83	0.373^b^
FIN (mU/L)	18.07 ± 16.31	17.27 ± 18.55	20.97 ± 15.56	0.089^b^
C-peptide (*μ*g/L)	3.41 ± 1.97	3.29 ± 2.03	3.83 ± 1.97	**0.013 ** ^b^
Hormonal variables				
24 h UFC (*μ*g)^#^	319.34 (167.30–613.94)	343.57 (159.43–670.08)	292.6 (172.32–586.8)	0.715^b^
Cortisol (*μ*g/dL)	26.72 ± 11.69	27.20 ± 11.24	26.37 ± 12.20	0.576^b^
TSH (mIU/L)^#^	1.05 (0.55–1.66)	0.97 (0.58–1.62)	1.09 (0.58–1.66)	0.323^b^
Low TSH, *N* (%)	71 (24.48)	47 (25.00)	24 (24.49)	0.998^a^
TT4 (pmol/L)	80.51 ± 21.16	79.75 ± 20.68	80.51 ± 22.24	0.854^b^
Low TT4, *N* (%)	32 (18.28)	19 (9.90)	13 (13.27)	0.234^a^
TT3 (pmol/L)	1.26 ± 0.45	1.21 ± 0.40	1.30 ± 0.44	0.328^b^
Low TT3, *N* (%)	151 (49.66)	106 (55.20)	45 (45.92)	0.134^a^
FT4 (pmol/L)	14.25 ± 2.88	14.48 ± 2.97	13.79 ± 2.63	**0.045 ** ^**b**^
Low FT4, *N* (%)	47 (16.20)	25 (13.02)	22 (22.45)	**0.040 ** ^a^
FT3 (pmol/L)	3.78 ± 0.95	3.73 ± 0.91	3.79 ± 0.91	0.757^b^
Low FT3, *N* (%)	115 (32.07)	79 (41.15)	36 (36.73)	0.468^a^
FT3/FT4	0.27 ± 0.07	0.26 ± 0.07	0.28 ± 0.06	**0.037 ** ^b^

^a^Pearson chi-squared test. ^b^Student's t-test. ^#^Log transformed before analysis. BMI: body mass index; IGT: impaired glucose tolerance; TSH: thyroid stimulating hormone; TT4: total thyroxine; TT3: total triiodothyronine; FT4: free thyroxine; FT3: free triiodothyronine; AST: aminotransferase; ALT: alanine aminotransferase; GGT: gamma-glutamyl transferase; PLT: platelets; TC: cholesterol; TG: triglycerides; HDL-c: high density lipoprotein-cholesterol; LDL-c: low density lipoprotein-cholesterol; HbA1c: glycated hemoglobin; FBG: fasting blood glucose; FIN: fasting insulin; 24 h UFC: 24 hours urine cortisol. Reference range: TSH (0.550 ~ 4.780 mIU/L), TT4 (54.0‐174.0 pmol/L), TT3 (1.23‐3.39 pmol/L), FT4 (11.5‐22.7 pmol/L), and FT3 (3.50‐6.50 pmol/L). *P* value < 0.05 is shown in bold.

**Table 2:** Univariate and multivariate logistic regression analysis for the associations of MAFLD with hormones and the presence of metabolic syndrome components in patients with Cushing's disease.

	Univariate model		Multivariate model 1		Multivariate model 2	
	OR (95% CI)	*P* value	OR (95% CI)	*P* value	OR (95% CI)	*P* value
Age	1.001 (0.982–1.020)	0.915	1.002 (0.978–1.028)	0.850	0.985 (0.957–1.014)	0.319
BMI	1.228 (1.189–1.395)	**<0.001**	1.315 (1.197–1.444)	**<0.001**	1.313 (1.192–1.447)	**<0.001**
Sex	1.794 (1.010–3.177)	**0.045**	2.705 (1.229–5.955)	**0.013**	2.227 (0.986–5.033)	0.054
Diabetes/IGT	1.340 (0.812–2.208)	0.252	—	—	1.343 (0.666–2.706)	0.410
Hypertension	2.444 (1.321–4.522)	**0.004**	—	—	2.610 (1.114–6.116)	**0.027**
Hyperlipidemia	2.283 (1.357–3.842)	**0.002**	—	—	1.661 (0.836–3.300)	0.147
Central hypothyroidism	0.611 (0.321–1.161)	0.132	—	—	—	—
Cortisol	0.994 (0.973–1.015)	0.574	0.995 (0.963–1.028)	0.755	0.988 (0.955–1.022)	0.470
24 h UFC^#^	1.000 (1.000–1.000)	0.525	1.000 (1.000–1.000)	0.462	1.000 (1.000–1.000)	0.409
TSH^#^	1.101 (0.871–1.394)	0.421	1.284 (0.842–1.959)	0.245	1.260 (0.820–1.938)	0.292
TT4	1.001 (0.990–1.013)	0.854	1.025 (0.997–1.054)	0.078	1.026 (0.997–1.055)	0.077
TT3	1.306 (0.765–2.230)	0.329	1.021 (0.209–4.993)	0.980	1.132 (0.222–5.766)	0.881
FT4	0.974 (0.896–1.058)	0.055	0.782 (0.647–0.946)	**0.011**	0.770 (0.633–0.937)	**0.009**
FT3	1.042 (0.806–1.346)	0.756	0.658 (0.332–1.305)	0.231	0.654 (0.330–1.298)	0.225

Multivariate model 1: The logistic regression analysis included age, BMI, sex, cortisol, 24h UFC, TSH, TT4, TT3, FT4, and FT3. Multivariate model 2: The logistic regression analysis included age, BMI, sex, diabetes/IGT, hypertension, hyperlipidemia, cortisol, 24h UFC, TSH, TT4, TT3, FT4, and FT3. BMI: body mass index; IGT: impaired glucose tolerance; 24 h UFC: 24 hours urine cortisol; TSH: thyroid stimulating hormone; TT4: total thyroxine; TT3: total triiodothyronine; FT4: free thyroxine; FT3: free triiodothyronine. *P* value < 0.05 is shown in bold.

**Table 3:** Multivariate logistic regression analyses the associations of quartiles of thyroid hormones and MAFLD in patients with Cushing's disease.

	Multivariate model	
	OR (95% CI)	*P* value
Age	0.990 (0.963–1.018)	0.496
BMI	1.316 (1.199–1.446)	**<0.001**
Sex	1.751 (0.810–3.783)	0.154
Cortisol	0.988 (0.958–1.019)	0.452
24 h UFC	2.465 (1.130–5.381)	0.453
Diabetes/IGT	1.753 (0.931–3.301)	0.082
Hypertension	2.610 (1.116–6.062)	**0.027**
Hyperlipidemia	1.016 (0.994–1.039)	0.160
TSH quartiles		0.248^*#*^
Q1	Ref	—
Q2	0.551 (0.199–1.522)	0.250
Q3	1.413 (0.552–3.615)	0.471
Q4	1.181 (0.423–3.298)	0.751
TT4 quartiles		0.202^*#*^
Q1	Ref	—
Q2	0.856 (0.307–2.389)	0.767
Q3	2.411 (0.764–7.608)	0.133
Q4	2.376 (0.568–9.940)	0.236
TT3 quartiles		0.999^*#*^
Q1	Ref	—
Q2	1.054 (0.366–3.034)	0.992
Q3	1.013 (0.274–3.745)	0.985
Q4	0.986 (0.187–5.180)	0.986
FT4 quartiles		**0.018 ** ^*#*^
Q1	Ref	—
Q2	0.945 (0.362–2.466)	0.908
Q3	0.257 (0.089–0.742)	**0.012**
Q4	0.220 (0.059–0.824)	**0.025**
FT3 quartiles		0.556^*#*^
Q1	Ref	—
Q2	0.541 (0.195–0.502)	0.239
Q3	0.796 (0.242–2.624)	0.708
Q4	0.470 (0.099–2.226)	0.341

BMI: body mass index; IGT: impaired glucose tolerance; 24 h UFC: 24 hours urine cortisol; TSH: thyroid stimulating hormone; TT4: total thyroxine; TT3: total triiodothyronine; FT4: free thyroxine; FT3: free triiodothyronine. ^#^*p*-value for trend. *P* value < 0.05 is shown in bold.

## Data Availability

The data used to support the findings of this study are available from the corresponding author upon reasonable request.
